# High expression of LAMA3/AC245041.2 gene pair associated with KRAS mutation and poor survival in pancreatic adenocarcinoma: a comprehensive TCGA analysis

**DOI:** 10.1186/s10020-021-00322-2

**Published:** 2021-06-16

**Authors:** Chengming Tian, Xiyao Li, Chunlin Ge

**Affiliations:** Department of Hepatopancreatobiliary Surgery, The First Hospital of China Medical University, Shenyang, 110000 Liaoning People’s Republic of China

**Keywords:** Pancreatic adenocarcinoma, KRAS, WGCNA, mRNA, lncRNA, Prognosis model

## Abstract

**Background:**

Pancreatic adenocarcinoma (PAAD) is one of the most challenging cancers with high morbidity and mortality. KRAS mutations could occur as an early event in PAAD. The present study aimed to identify the differentially expressed lncRNAs (DE-lncRNAs) and differentially expressed mRNAs (DE-mRNAs) in KRAS-mutant PAAD to explore the pathogenesis and the underlying molecular mechanism of PAAD development.

**Methods:**

Clinical data of TCGA–PAAD patients were downloaded from the TCGA database and subjected to survival analysis along with the KRAS mutation information data. Weighted gene correlation network analysis (WGCNA) and univariate Cox regression analysis were conducted to construct prognostic risk models to identify the hub DE-mRNAs and DE-lncRNAs associated with PAAD prognosis. GO and KEGG enrichment analyses of the identified hub DE-mRNAs were performed. Multivariate cox regression analysis was performed to analyze the overall prognosis of age, gender, pathologic_T, and KRAS mutations, following which the differences in the clinical characteristics of risk score1 and risk score2 were analyzed. Finally, the mRNAs–lncRNA–TFs regulatory network was constructed.

**Results:**

Functional enrichment analysis was performed after screening 1671 DE-mRNAs and 324 DE-lncRNAs. It was observed that the associated pathways were enriched mainly in the modulation of chemical synaptic transmission, synaptic membrane, ion-gated channel activity, ligand−receptor interactions that stimulate neural tissue, among others. The univariate Cox regression analysis screened 117 mRNAs and 36 lncRNAs, and the risk ratio models of the mRNAs and lncRNAs were constructed. LAMA3 (mRNA) and AC245041.2 (lncRNA) exhibited a strong expression correlation in the respective two risk models. The genes in the samples with a high expression of these two genes were enriched in several pathways associated with transcription factors (TFs), among which the TFs ATF5, CSHL1, NR1I2, SIPA1, HOXC13, HSF2, and HOXA10 were shared by the two groups. The core enrichment genes in the common TF pathways were collated, and the mRNAs–lncRNAs–TFs regulatory network was constructed.

**Conclusion:**

In the present study, novel prognostic mRNAs and lncRNAs were identified, and their respective prognostic models and nomograms were constructed to guide clinical practice. An mRNAs–lncRNAs–TFs regulatory network was also constructed, which could assist further research in the future.

**Supplementary Information:**

The online version contains supplementary material available at 10.1186/s10020-021-00322-2.

## Introduction

Pancreatic adenocarcinoma (PAAD) is a malignant tumor that occurs in the exocrine glands of the pancreas. Pancreatic malignancies may originate from the exocrine, endocrine, or non-epithelial tissues of the pancreas, among which 95% of malignancies are pancreatic adenocarcinoma. PAAD has an extremely poor prognosis, with high morbidity and mortality. While the incidence and mortality of other common cancers have been decreasing in recent years, the mortality and number of deaths caused by pancreatic tumors have been increasing (Siegel et al. 2018; Rahib et al. 2014). Early surgery is the main treatment for PAAD; however, pancreatic adenocarcinoma is difficult to diagnose at an early stage, with most of it metastasized by the time of the initial diagnosis (DeSantis et al. 2014) and only 9.7% of the cases presenting localized PAAD at the time of diagnosis (National Cancer Institute 2018). Most deaths in PAAD cases occur due to liver, lung, and/or peritoneal metastasis, which are the most common sites of spread (Yachida and Iacobuzio-Donahue 2009). Moreover, PAAD does not respond well to most chemotherapy drugs (Vincent et al. 2011). Therefore, exploring the molecular mechanisms underlying the pathogenesis and development of PAAD is imperative.

Molecular biology studies have demonstrated that proto-oncogene activation, tumor suppressor gene inactivation, and abnormality in DNA repair genes are closely associated with the occurrence of PAAD (Roberts et al. 2016). Several important genes were observed to be mutated in PAAD, among which the mutation rate of P16 in PAAD patients was 95%, KRAS 90%, P53 75%, and DPC4 55% (Jones et al. 2008). KRAS is one of the most common mutant oncogenes in human cancers. Experiments in cell culture and animal models have confirmed that the development of several cancers relies on the sustained expression and signal transduction of KRAS (Haigis 2017; Hayes et al. 2016). McCormick F stated that KRAS targeting for cancer treatment was effective both when directly targeting the protein or using indirect approaches to target it, such as siRNA or harnessing the immune system (McCormick 2015). In recent years, gene profiling and next-generation sequencing technologies have become indispensable tools for cancer research as these enable the detection of cancer-associated genetic and epigenetic changes, such as mutations, copy number variations, and DNA methylation alterations across further extensive genomic regions (Huang et al. 2019; Stark et al. 2019). Bioinformatics analysis of these data might provide valuable information for PAAD research. For instance, Cheng synthesized several sets of public data and preliminarily elucidated the pathways and functions involved in pancreatic adenocarcinoma. Candidate molecular markers for the diagnosis and prognosis prediction of pancreatic adenocarcinoma were identified, and candidate proteins attributable to the clonal and invasive nature of pancreatic cancer cells were suggested (Cheng et al. 2019). Considering that KRAS mutation might occur as an early event in pancreatic adenocarcinoma, the present study began with KRAS mutation grouping, which was followed by the construction of prognostic models based on WGCNA to predict the prognosis of PAAD. Furthermore, a line map was constructed to guide clinical practice, while the hub lncRNAs and mRNAs in the constructed model were further analyzed to construct an mRNA–lncRNA–TFs regulatory network that would provide clues for subsequent research in this area.

## Materials and methods

### Data download

A workflow chart for the present study is provided in the Additional file 1.

PAAD expression data, clinical data, and phenotypic data were downloaded from the TCGA database.

Expression data: https://gdc.xenahubs.net/download/TCGA-PAAD.htseq_counts.tsv.gz

Clinical data: https://gdc.xenahubs.net/download/TCGA-PAAD.survival.tsv.gz

Phenotypic matrix:

https://gdc.xenahubs.net/download/TCGA-PAAD.GDC_phenotype.tsv.gz

Sample mutation data:

https://portal.gdc.cancer.gov/files/fea333b5–78e0–43c8-bf76–4c78dd3fac92

Human.gtf file from the Ensembl database (Homo_sapiens.GRCh38.99.gtf.gz), lncRNAs, and symbol information:

http://www.ensembl.org/info/data/ftp/index.html

### Survival analysis

KRAS mutation information was extracted from the mutation data of PAAD samples. Phenotypic data were integrated and grouped based on whether KRAS was mutated or not. Next, a KM curve was plotted, and the P-value of the curve was determined to be less than 0.05, indicating a significant survival difference among the groups.

### Screening for differentially expressed lncRNAs and mRNAs

R package “edgeR” was employed to identify the differentially expressed genes (DEGs). The expression matrix in the database was in the form of log2(count 1); therefore, round(2^a-1) was used for obtaining the counts of the sample. Subsequently, the low-expression genes were filtered based on the criterion of CPM (count-per million) being greater than 1 in at least 10 samples, and the differentially expressed genes were extracted using the thresholds of |logFC|> 1 and FDR < 0.05. Next, the standardized expression matrix was extracted and used as the expression spectrum in the subsequent analysis. Finally, the genetic information was obtained from the human.gtf files, and DE-mRNAs and DE-lncRNAs were extracted for subsequent analysis.

### Functional enrichment analysis

First, R package “clusterProfiler” was employed to perform the functional enrichment analysis of differential DE-mRNAs (Yu et al. 2012; Kanehisa et al. 2010), with P-value < 0.05 and Q-value < 0.2 as the screening thresholds. After obtaining the enrichment pathway, results were visualized using the R package “GOplot”.

### Weighted gene co-expression network analysis (WGCNA)

The weighted gene co-expression network analysis aimed to identify the co-expression gene modules, explore the association between the gene networks and the phenotypes of concern, and identify the hub genes in the network. The main principle is to use the correlation coefficient of the expression quantity between the genes to the power of n, and the direct result is the amplification of the difference of the correlation between the genes. A specific value β was used as the power of the correlation coefficient between each pair of genes (i, j) to calculate the correlation among all genes, that is, the adjacency matrix: a_i, j_ =|cor (i,j) |^β^. In order to better determine whether two genes have similar expression profiles, WGCNA adopts a method based on the soft threshold. Since the result of adjacency (a_i,j_) relies directly on the value of β, which directly affects the construction of the module and division of the adjacent genes, WGCNA calculates the β value according to the adjacent lowest value of the scale-free network. A scale-free network is characterized by a small number of nodes, with degrees significantly higher than the general points, which are referred to as hubs. A few hubs are associated with other nodes and ultimately constitute the entire network. When β value was selected for network construction, network construction and module identification were conducted in the following four steps: the similarity between each gene was calculated through topological overlap; the gene cluster tree was obtained; genes with the same expression were classified into the same module by cutting the tree; similar modules were merged, and after the module classification was completed, the correlation between the different modules and phenotypes was determined, and the more relevant modules were used for subsequent analysis. The R package “WGCNA” was employed to analyze the weighted co-expression network of all the differentially expressed genes (Langfelder and Horvath 2008), and the module with the strongest correlation with the prognostic traits was subjected to subsequent analysis.

### Univariate Cox regression analysis

In order to deeply explore the genes associated with prognosis in the differentially expressed genes and survival data, the R-package “survival” and “survminer” tools were employed for conducting batch univariate Cox regression analysis. After the regression analysis, the significantly correlated genes were screened using the P < 0.05 threshold and used for the subsequent model construction. Among these genes, the top 6 genes were selected for Kaplan–Meier analysis.

### Construction of the prognostic risk model

The mRNAs and lncRNAs identified in the Cox regression results were subjected to LASSO regression dimensionality reduction, and a risk scoring model was constructed, which mainly relied on the R package “glmnet” tool. In order to construct a further accurate regression model, lambda screening was first conducted using cross-validation, and then the corresponding model of lamdba.min was selected to extract further the expression matrix of the relevant genes in the model. The risk score of each sample was calculated based on the following formula:

where exp represents the expression level of the corresponding gene, β_j_ is the

$${\text{RScore}}_{{\text{i}}} = \sum\limits_{{{\text{j}} = 1}}^{{\text{n}}} {\exp_{{{\text{ji}}}} \times {\upbeta }_{{\text{j}}} }$$

regression coefficient (coef) of the corresponding gene in the multivariate regression results, RScore equals the expression level of the significantly related gene in each sample multiplied by the coef of the corresponding gene, summed over, i represents the sample, and j represents the gene. According to the risk scores, the samples were classified into high and low-risk groups by the median of the nodes to conduct the subsequent model performance evaluation.

After the high and low-risk groups were obtained, Kaplan–Meier analysis was conducted on these groups and the survival data. Subsequently, the ROC curve was drawn using the sample risk scores as the model prediction results. The AUC value was greater than 0.6, indicating that the model exhibited good performance.

### Multivariate Cox regression analysis and nomogram construction

In order to verify the mRNA and lncRNA prognostic model as an independent prognostic factor for the disease, Cox multivariate regression analysis was performed to analyze the overall prognosis of age, gender, pathologic_T, and KRAS mutations.

A nomogram was used for visualizing the results of Cox regression analysis. Nomogram sets the scoring standard according to the regression coefficient of all the independent variables, assigns a score to each level of value for each independent variable, calculates a total score for each patient, and also calculates the probability of the outcome time for each patient through the conversion function between the score and the probability of the outcome.

The nomogram was constructed using the R package “rms” and “survival” tools mainly. First, the scale risk regression model was constructed using cph(), followed by calculating the survival probability using the survivalcph() function. Finally, the nomogram object was constructed using the nomogramcph() function and displayed using plotcph().

### The difference analysis of the clinical characteristics of risk score1 and risk score2

The clinical indicators, namely, age, gender, pathological_M, pathological_N, pathological_T, and Tumor_stage, were selected to detect differences between risk score1 and risk score2 in these indicators. The “ggpubr” package was employed to draw the boxplot representing the results. Afterward, the difference in the distribution within the group was further detected using the t-test to verify whether the risk score was consistent with the clinical indicators.

### Analysis of important regulatory relationships in the risk prognostic models

The mRNAs and lncRNAs demonstrating the strongest correlation with the prognostic traits were obtained from the two models, respectively. The KRAS mutation samples were extracted and grouped based on the expression of hub mRNAs and lncRNAs, respectively. Subsequently, the KM curve was drawn to explore the respective relationships of the expressions of hub mRNAs and lncRNAs with the prognostic traits. Afterward, the expression levels of the hub mRNAs and lncRNAs were used for predicting the prognosis of the samples, and the ROC curve was drawn. Finally, multivariate COX regression analysis was conducted in combination with the clinical phenotypes to verify the independent prognostic efficacy of the hub mRNAs and lncRNAs.

Furthermore, the GSEA analysis of the hub mRNAs and lncRNAs was performed (Subramanian et al. 2005; Mootha et al. 2003). The nodes were divided into high and low expression groups based on the median expression of the hub mRNAs and lncRNAs, and subsequently, the GSEA analysis was conducted. The results were filtered using the thresholds of P-value < 0.05 and FDR < 0.25.

### Construction of the TF regulation network associated with the hub mRNA–lncRNA regulatory axis

On the basis of the expression levels of hub mRNAs and lncRNAs, the differentially expressed genes were extracted using edgeR, and the core genes enriched into the TF pathways according to the key single gene GSEA were integrated to construct the mRNAs–lncRNAs–TFs regulatory network.

## Results

### Data download

PAAD-related expression data and clinical data were downloaded from the UCSC TGCA database. The samples with missing clinical information were removed, the samples with the KRAS mutation phenotype were integrated, and finally, the data for 177 cancer samples were obtained, which included 128 KRAS mutation samples and 49 non-mutation samples (Table 1).

**Table 1:** Phenotype statistics of TCGA–PAAD

	Tumor
KRAS	
Yes	128
No	49
Gender	
Male	97
Female	80
Age (mean/median = 65 years)	
> 65	84
≤ 65	93
Tumor_stage	
Stage I	24
Stage II	34
Stage III	35
Stage IV	17
Pathologic_M	
M0	79
M1	5
MX	93
Pathologic_N	
N0	50
N1	122
NX	4
Pathologic_T	
T1	7
T2	24
T3	141
T4	3
TX	1

### Survival analysis

**Fig. 1:**
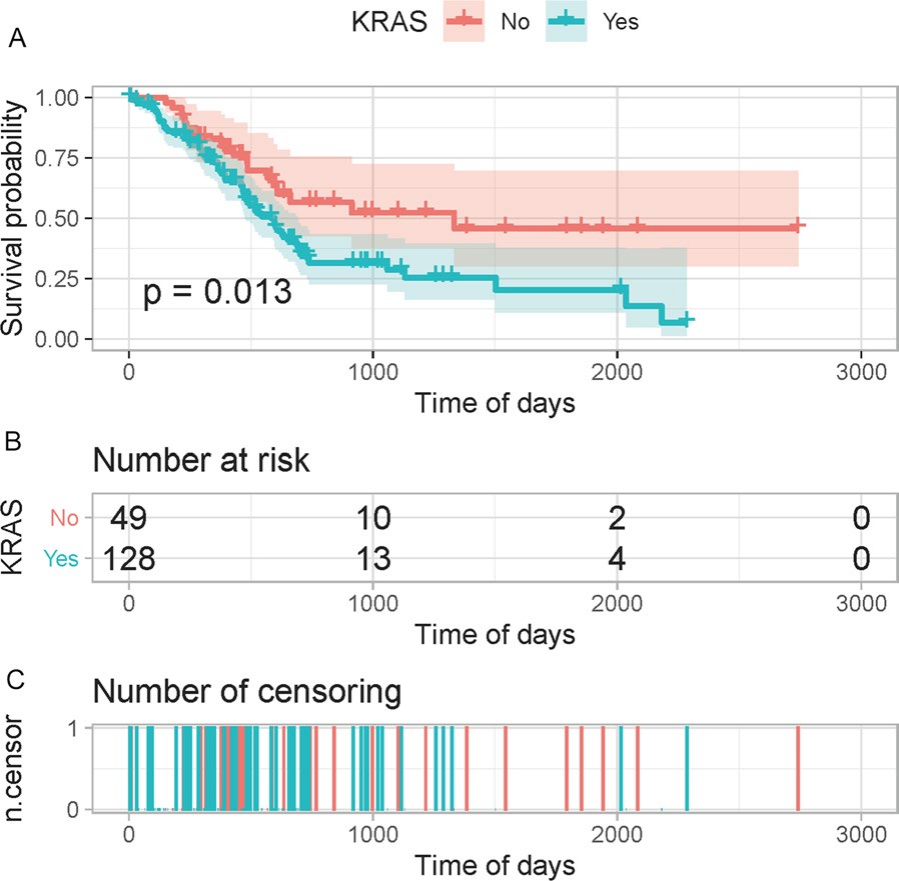
Survival analysis of KRAS mutation: **A** survival curve. **B** Number of individuals at risk (possibility of death) over time in both groups of samples. **C** Number of dead patients at each time point

According to the presence or absence of KRAS mutation, the samples were divided into two groups and then subjected to survival analysis. The KM curve revealed that the survival difference between the two groups was significant (P = 0.013), and the survival curve of the KRAS mutation samples declined faster (Fig. 1). In addition, it was confirmed that the survival rate of patients in the KRAS-mutant group was significantly lower than that in the KRAS-wildtype group.

### DE-mRNAs and DE-lncRNAs between KRAS-mutant and KRAS-wildtype PAAD

**Fig. 2:**
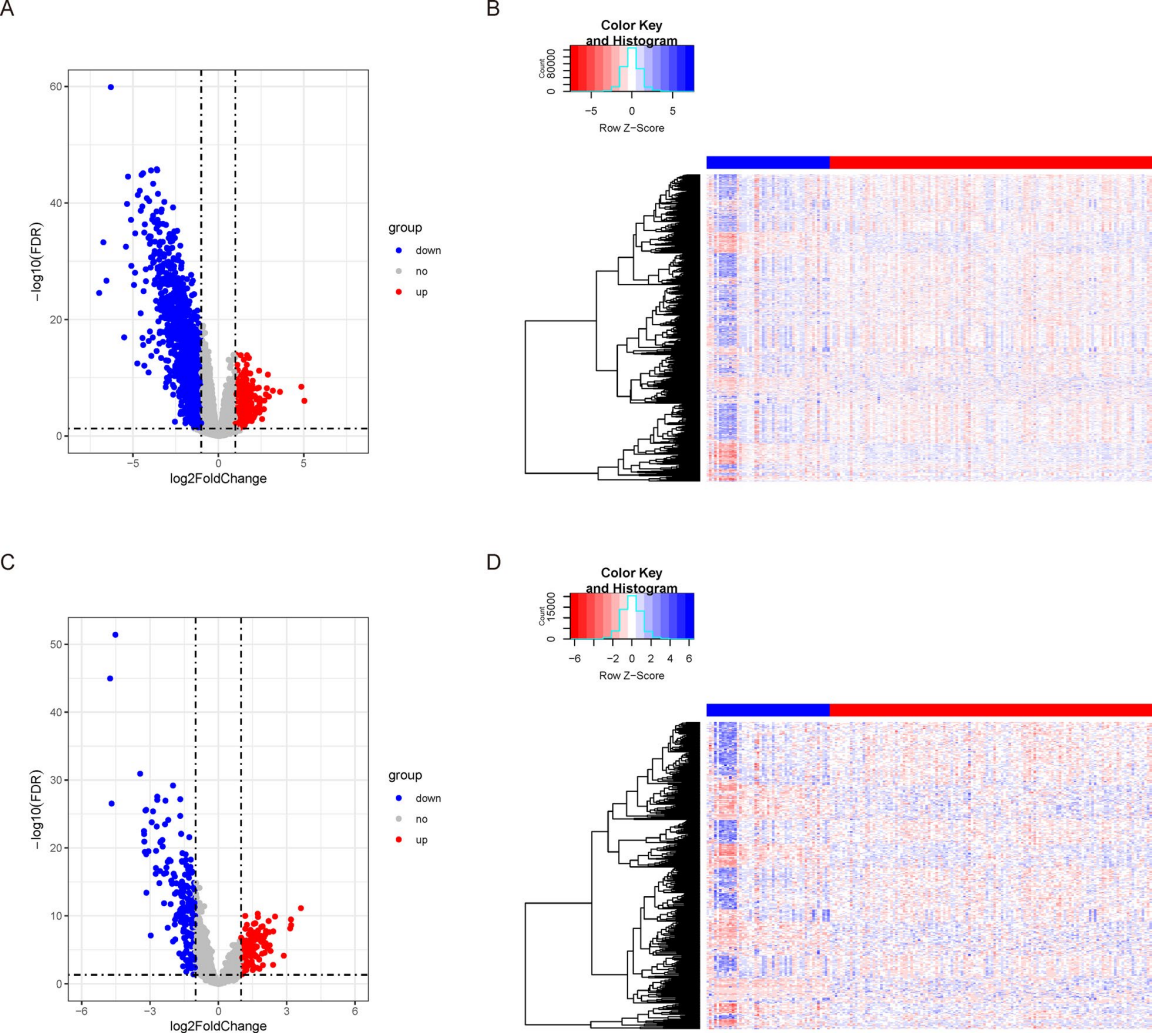
Statistics on differential expression. Volcano plot for differential gene expression, with the upregulated genes represented by red and the downregulated genes represented by blue (**A**: mRNAs; **C**: lncRNAs). Heat map for differential gene expression, with the red legend depicting the mutated samples, the blue legends depicting the non-mutated samples, and the decrease of expression demonstrated from blue to red (**B**: mRNAs; **D**: lncRNAs)

In order to further explore the KRAS mutation-related mRNAs and lncRNAs in PAAD, the differential expressions of lncRNAs and mRNAs between patients of the KRAS-mutant group and those of the KRAS-wildtype group were analyzed. The DE-mRNAs and DE-lncRNAs were obtained from the expression data of the TCGA–PAAD cancer samples using the screening thresholds |logFC|> 1 and FDR < 0.05, which revealed 1671 DE-mRNAs (368 upregulated and 1302 downregulated) and 324 DE-lncRNAs (56 upregulated and 171 downregulated) (Fig. 2).

### DE-mRNA GO and KEGG enrichment analyses

**Fig. 3:**
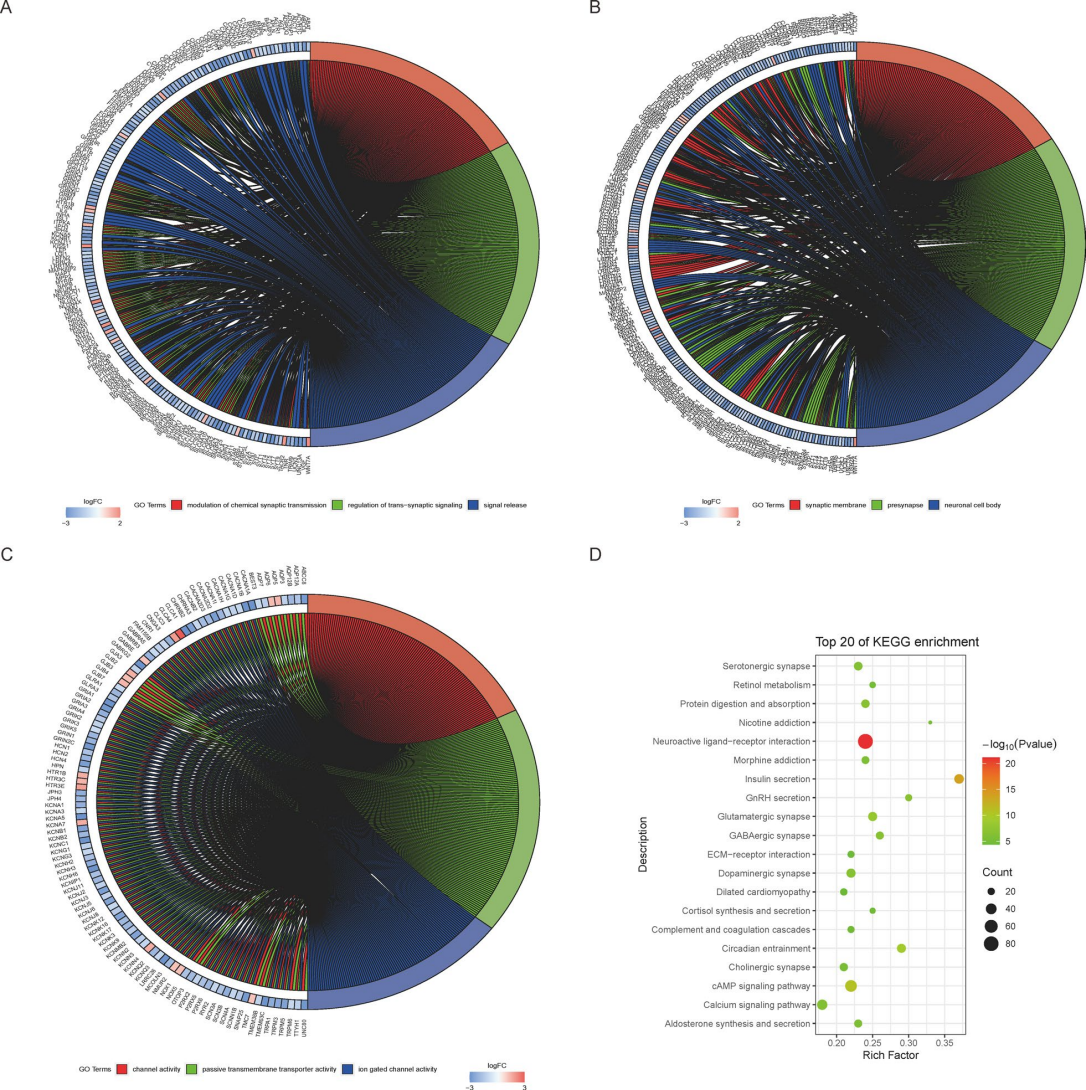
**A**–**C** The results of functional enrichment analysis, which included three types of GO analysis: the enrichment pathways with the highest P-value are on the left, and the corresponding genes of the enrichment pathways are on the right. Log FC represents the differential expression multiple of these genes. **D** KEGG analysis: The higher intensity of red represents higher significance. The larger the dot, the more genes enriched in that pathway

The functional enrichment analysis of the identified DE-mRNAs was conducted. The GO enrichment analysis had the following three aspects: Biological Process (BP), Cell Components (CC), and Molecular Function (MF). The BP enrichment pathway mainly involved the modulation of chemical synaptic transmission and the regulation of trans-synaptic signaling and signal release. The CC enrichment pathway mainly involved synaptic membrane, neuronal cell body, etc. The MF enrichment pathway mainly involved channel activity, passive transmembrane transporter activity, and ions gated channel activity, etc. The pathways of KEGG enrichment mainly involved neuroactive ligand-receptor interaction, insulin secretion, etc. (Fig. 3).

### WGCNA and identification of the prognosis-associated module

**Fig. 4:**
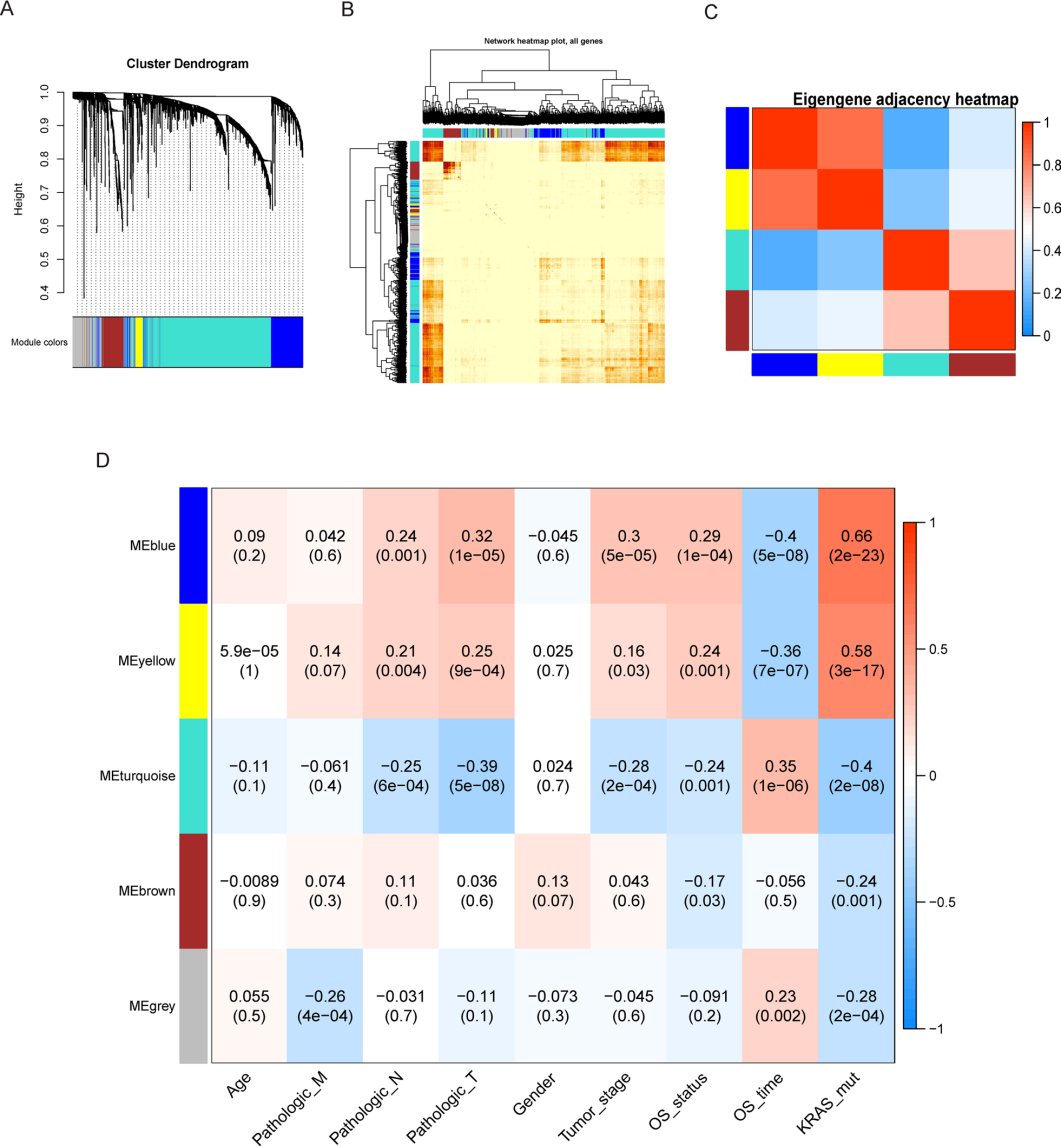
WGCNA analysis results. **A** Clustering results. Cluster dendrogram and the co-expression network modules identified in average linkage hierarchical clustering of DEGs based on topological overlaps. Each branch within the dendrogram represents a single gene. Height represents the Euclidean distance. Each color indicates a single module containing weighted co-expressed genes; **B** Weighted network heat map of all genes; **C** Eigengene adjacency heatmap. The heatmap illustrates the relationship among the distinctive co-expression modules; **D** Heat map of correlation between the modules and the phenotypes. Each row represents a color module, and each column indicates a clinical trait. Each cell contains the *R*2 values of Pearson’s correlations between the modules and the clinical features, and the corresponding *P*-values are inside the parentheses. The gradient color of each cell indicates the *R*2 values of Pearson’s correlations (red = 1, blue =  − 1). *DEGs* differentially expressed genes; *ME* module eigengene

WGCNA was performed with the DEGs, and prognosis-related modules were identified. First, the soft threshold was calculated; R^2 > 0.85 was used as the filtering threshold to obtain the power = 6. Subsequently, the network was constructed using a one-step method with power = 6, and similar modules were combined using the threshold of height < 0.25. Finally, four modules were obtained, among which the genes in the blue module (338) exhibited the highest correlation with the OS_status and OS_time parameters, and also with KRAS mutation. Therefore, the genes in this module were selected for subsequent analysis (Fig. 4).

### Univariate cox regression analysis

**Fig. 5:**
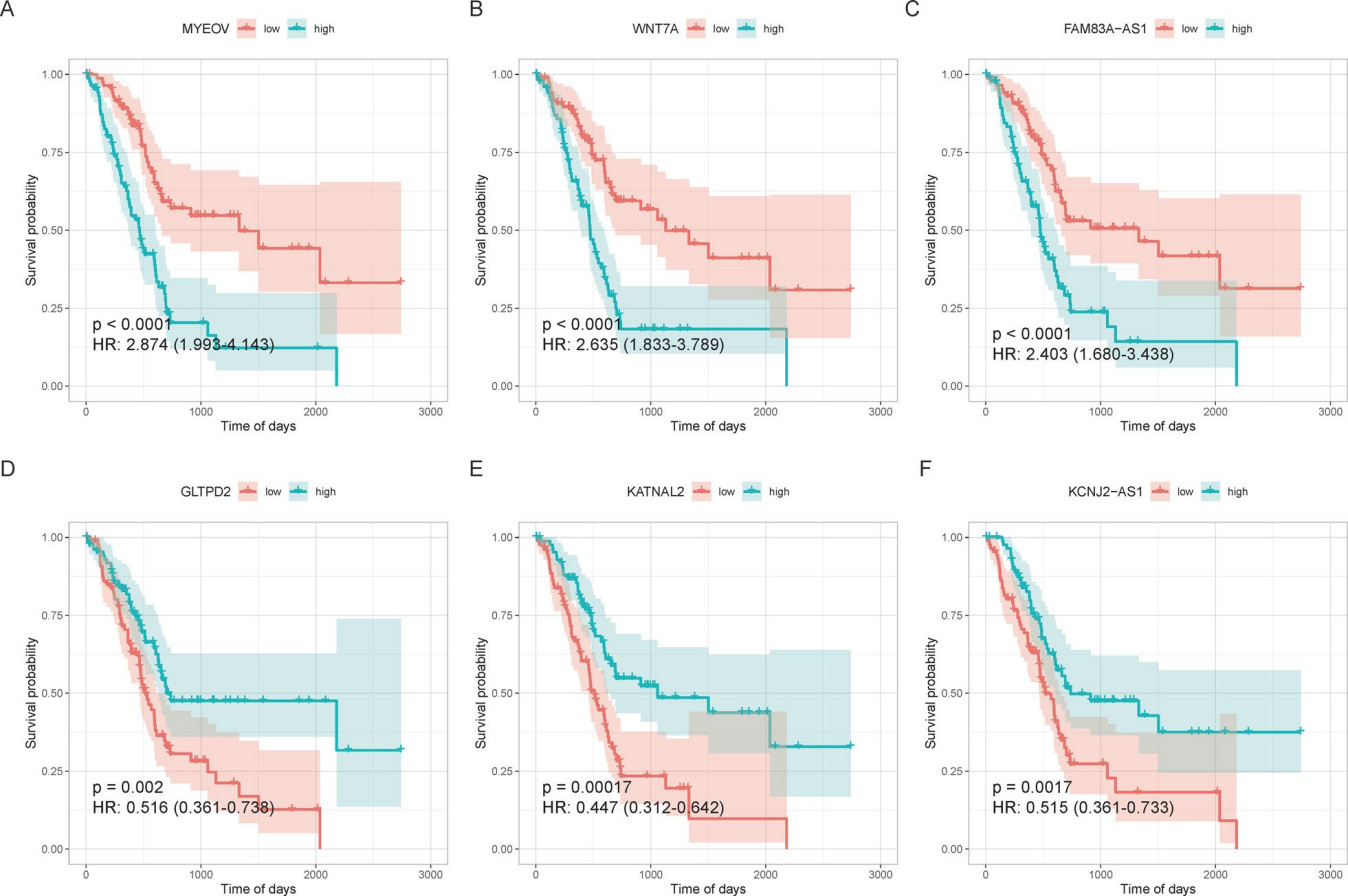
The KM curves of the top 6 genes exhibiting a significant correlation to disease prognosis

The differential expression matrix of 177 cancer samples was extracted for Cox regression analysis. After the screening based on the P-value, 153 genes significantly associated with PAAD were obtained, and the KM curve was drawn using the top 6 genes among these. The survival curves for the high-expression samples MYEOV, WNT7A, and FAM83A-AS1 declined rapidly, the hazard ratio was greater than 1, and the 95% CI was lower than 1, indicating that the high expression of these three genes might threaten survival. The survival curves for the high-expression samples KATNAL2, GLTPD2, and KCNJ2-AS1 declined gradually, the hazard ratio was greater than 1, and the 95% CI was also less than 1, indicating that a low expression of these three genes might threaten survival (Fig. 5).

### Construction of the mRNA and lncRNA prognostic risk model

**Fig. 6:**
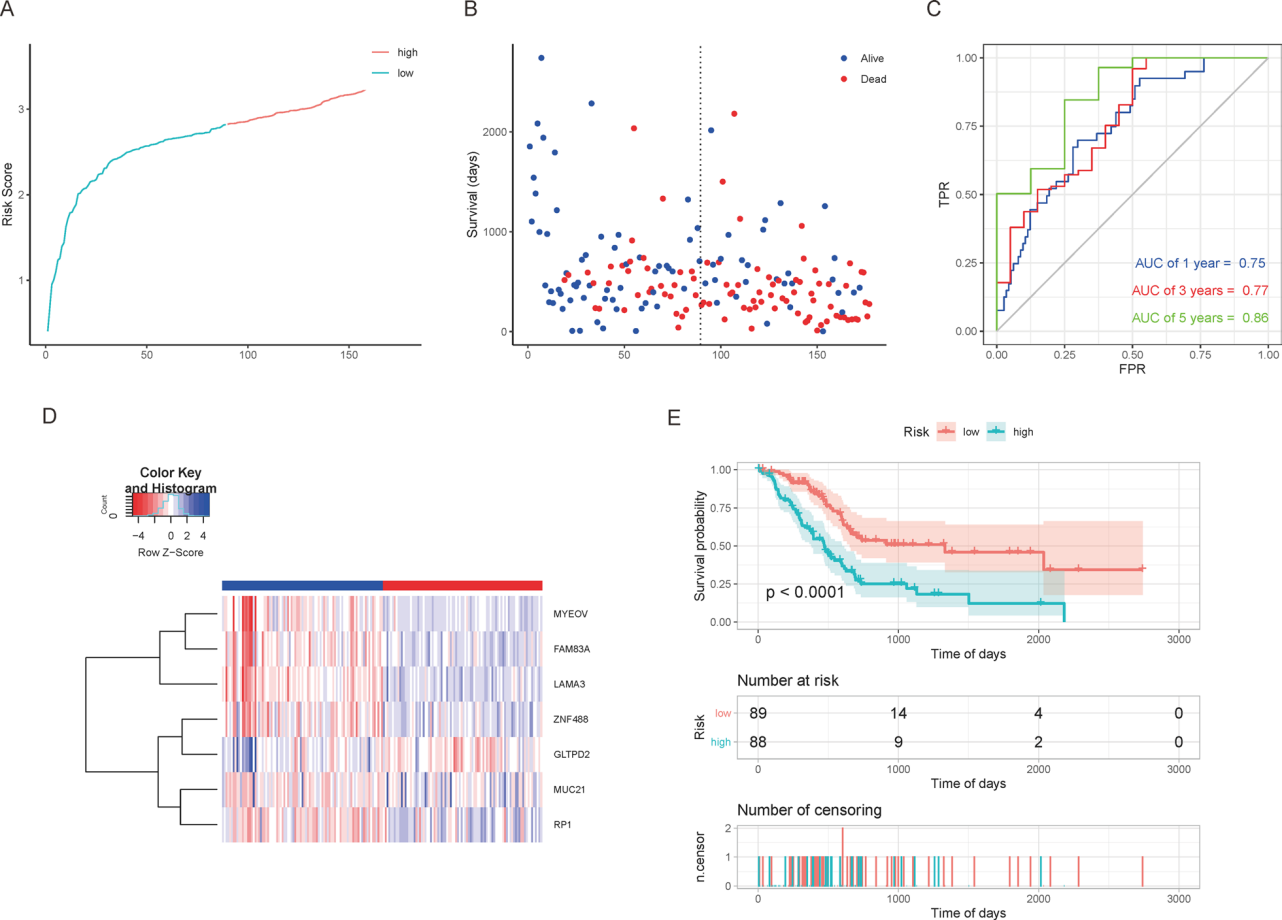
The mRNA prognostic risk model. **A** Sample risk score curve; **B** Scatter plot of sample survival time. Prior to the dotted line is the low-risk group sample, while the one after the dotted line is the high-risk group sample; **C** ROC curves depict the prognostic risk model; **D** Heat map of gene expression in the model. Values are normalized with log10. The right longitudinal axis: the names of mRNAs; the left longitudinal axis: the clustering information of the mRNAs. The upregulated and downregulated mRNAs are depicted in red and blue, respectively; **E** KM curves for high and low-risk groups

**Fig. 7:**
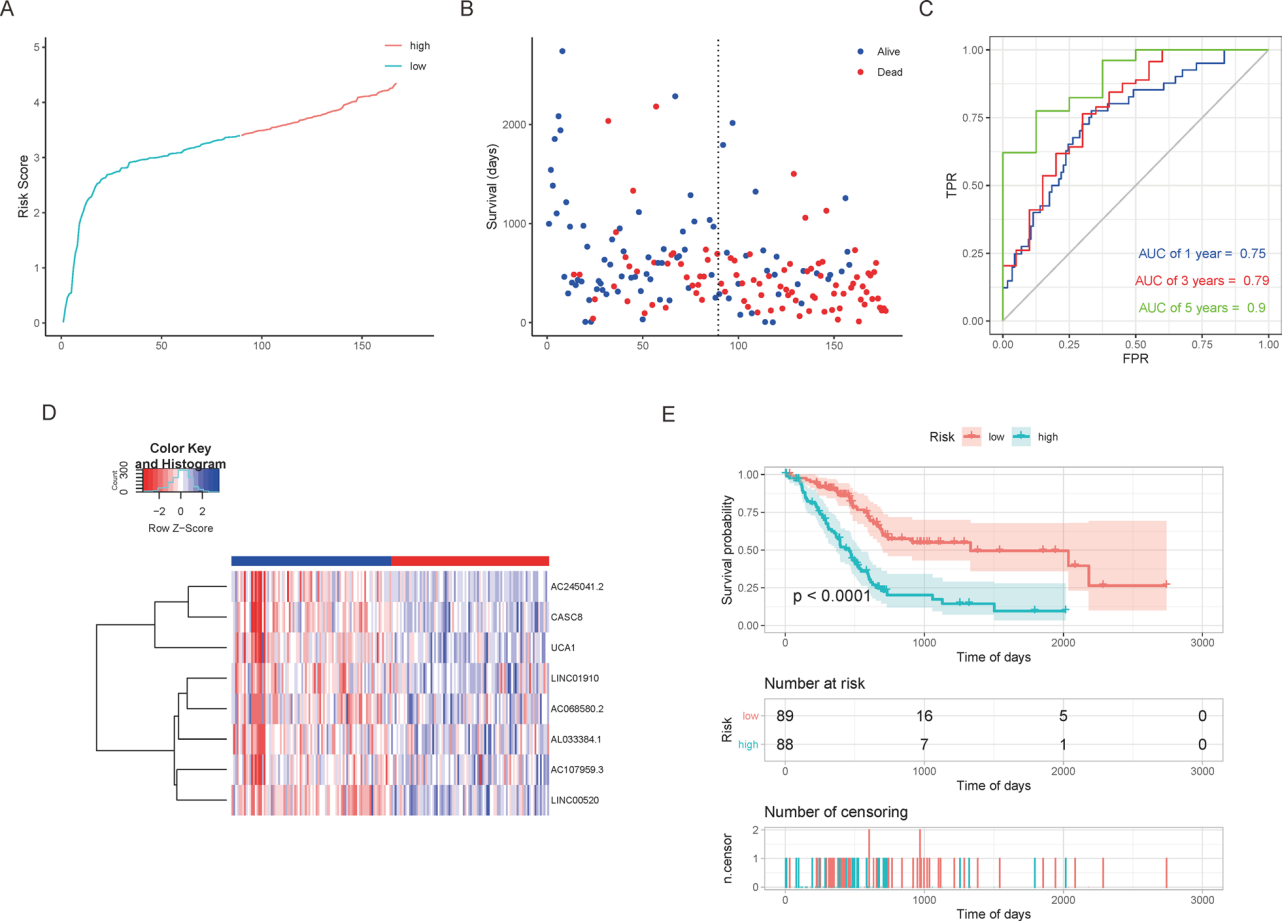
The lncRNA prognostic risk model: **A** Sample risk score curve; **B** Scatter plot of sample survival time. Prior to the dotted line is the low-risk group sample, and the one after the dotted line is the high-risk group sample; **C** ROC curves presenting the predictive values of the prognostic risk model; **D** Heat map of gene expression in the model. Values are normalized with log10. The right longitudinal axis: the names of lncRNAs; the left longitudinal axis: the clustering information of the lncRNAs. The upregulated and downregulated mRNAs are depicted in red and blue, respectively; **E** KM curves for high and low-risk groups

Seven mRNAs significantly associated with the prognosis of PAAD were screened out from 117 mRNAs using LASSO regression. Subsequently, a risk ratio model was constructed based on the expression of 7 markers and regression coefficients, that is, risk score1 = GLTPD2*(− 0.113) + RP1 × 0.008 + MUC21 × 0.016 + FAM83A*0.029 + MYEOV*0.035 + ZNF488 × 0.083 + LAMA3 × 0.153. Eight lncRNAs significantly associated with the prognosis of PAAD were screened out from 36 lncRNAs using LASSO regression. Subsequently, a risk ratio model was constructed based on the expression of 8 markers and regression coefficients, that is, risk score2 = AC068580.2 × 0.003 + LINC01910 × 0.035 + AC245041.2 × 0.044 + AC107959.3 × 0.058 + CASC8 × 0.100 + UCA1 × 0.102 + LINC00520 × 0.126 + AL033384.1 × 0.170. Afterward, the risk scores of all samples were calculated, and based on the median; the samples were divided into high and low-risk groups; the KM curve for these high and low-risk groups was then drawn. The results demonstrated that the difference between the high and low-risk groups was significant (P < 0.0001) and that the AUC values for 1 year, 3 years, and 5 years in the ROC curve were greater than 0.75, which indicated that the mRNA and lncRNA model has good prediction efficiency (Figs. 6 and 7).

### Multivariate cox regression analysis and nomogram construction

**Fig. 8:**
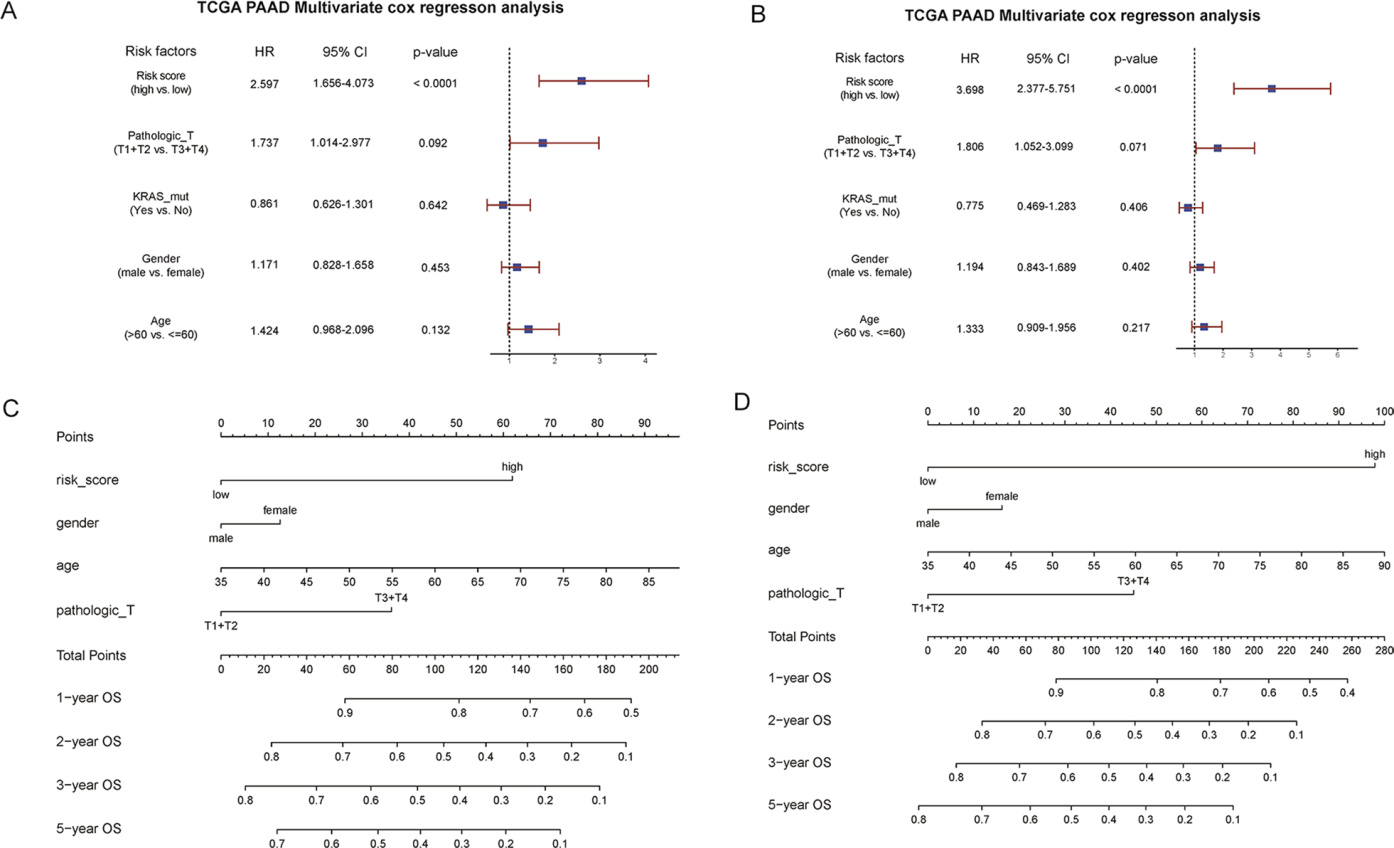
(**A** mRNA, **B** lncRNA) Results of multivariate Cox regression analysis. Forest plot of the multivariable Cox regression analysis. The squares on the transverse lines present the hazard ratio (HR), and the transverse lines represent the 95% confidence interval (CI); (**C** mRNA, **D** lncRNA) nomogram of the overall survival prediction in PAAD

In combination with age, gender, pathologic_T, and KRAS mutations, Cox multivariate regression was used for verifying the mRNA and lncRNA prognostic model. Multivariate cox regression results demonstrated that the risk score1 (2.597) and risk score2 (3.698) had the highest HR, and pathologic_T also had better predictive efficacy. However, the predictive efficacies of age, gender, and KRAS mutation were poor. HR was < 1, and P-value was not significant in the multivariate regression analysis, indicating that the presence or absence of KRAS mutation was not suitable for use as a single factor for prognostic analysis. Therefore, the factor was removed in the subsequent construction of the nomogram. In the constructed nomogram, the high risk, high age, and high pathologic_T grade samples scored higher and presented a higher survival risk (Fig. 8).

### Difference analysis of the clinical characteristics of risk score1 and risk score2

**Fig. 9:**
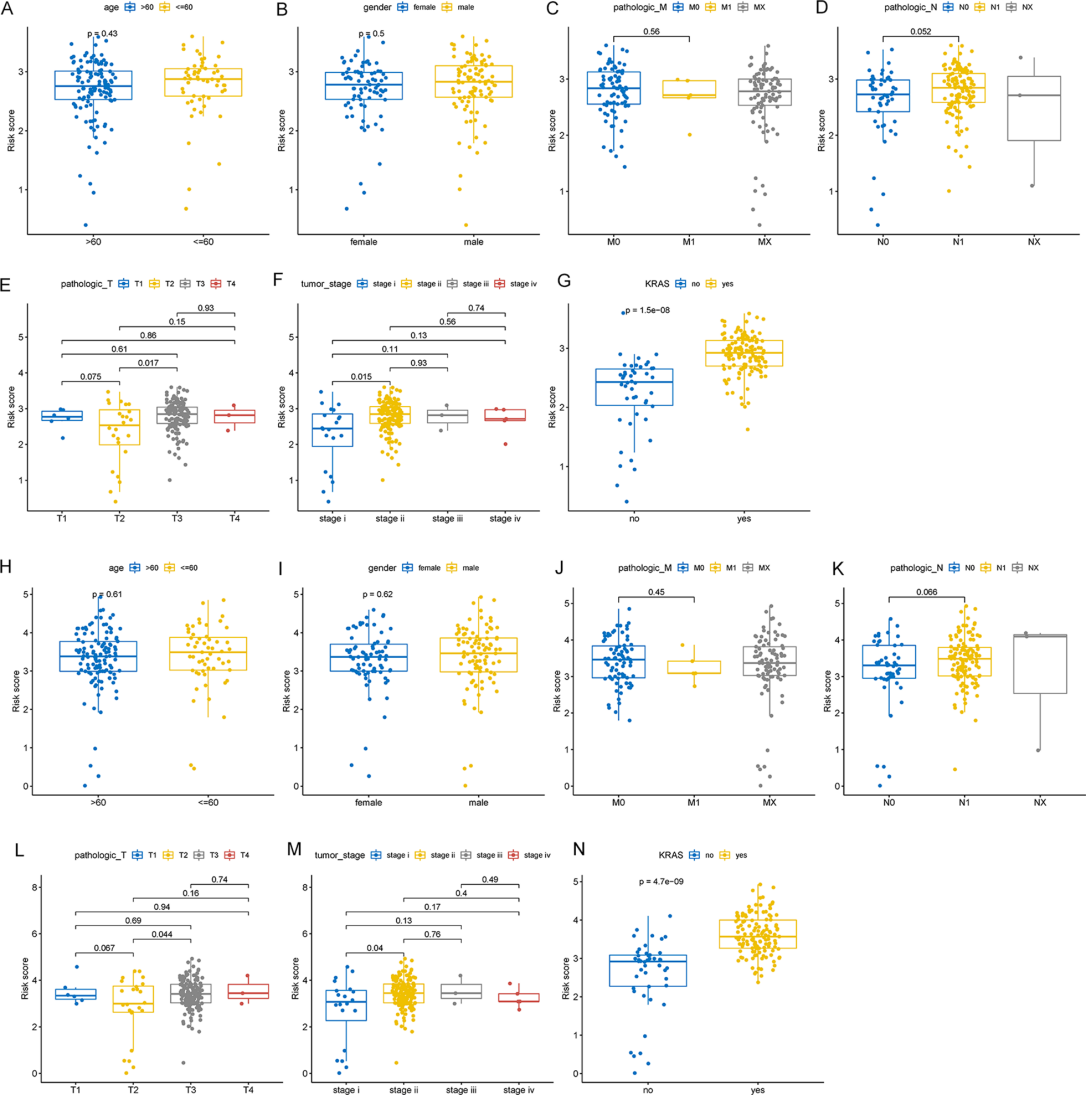
**A**–**G** Distribution of risk score1 in the clinical phenotypes. **H**–**N** Distribution of risk score2 in the clinical phenotypes

As depicted in Fig. 9, the risk scores of the two models were significantly different in different KRAS mutation states, with the risk scores of patients with KRAS mutation being generally higher, indicating that our models exhibited a close association with KRAS mutation. Further observation of the difference in the risk scores within the Pathological_T group revealed that the small number of T1 and T4 patients rendered the statistical results meaningless, while the risk score of T2 patients was significantly lower than that of T3 patients, indicating that the prediction results of the two models were in good agreement with the diagnosis of patients in the Pathological_T group. Finally, the difference in the risk score within the tumor stage group was analyzed. Since the number of Stage III and Stage IV patients was extremely small, the statistical results were rendered meaningless, and the analysis was, therefore, focused on Stage I and Stage II patients. It was observed that the risk score of Stage II patients was significantly higher than that of Stage I patients, indicating that risk score and tumor stage were consistent with each other. Therefore, it was concluded that the risk score predictions of the two models were consistent with the clinical diagnosis results as well as with the KRAS mutation results, which further reinforced the good accuracy of our two models.

### Important regulatory relationships in risk-prognostic models

**Fig. 10:**
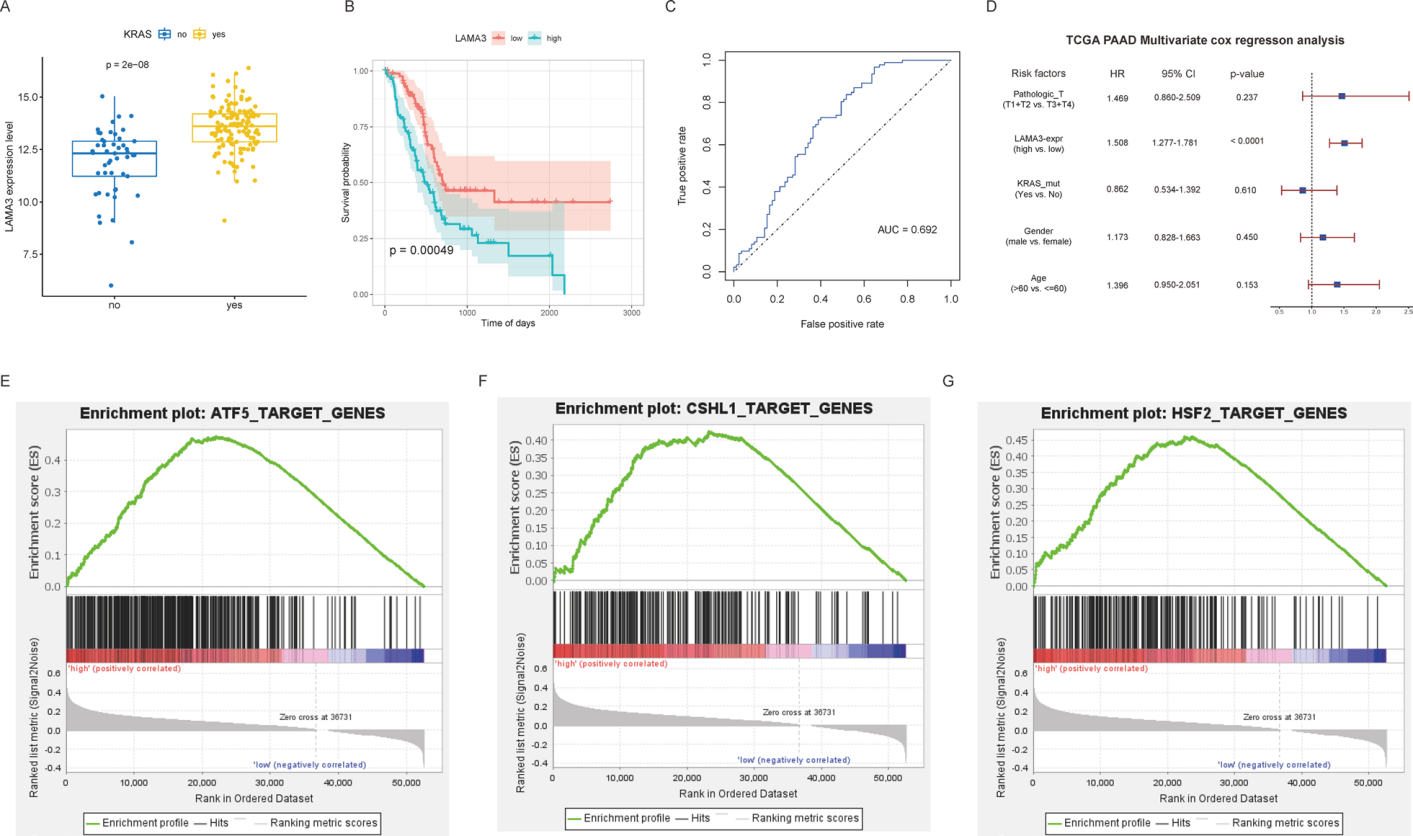
**A** Distribution of LAMA3 expression in KRAS mutation groups. **B** KM curves to compare the overall survival between high-LAMA3 and low-LAMA3 samples. **C** ROC curve for prognosis based on LAMA3 expression. **D** Multivariate Cox regression analysis based on LAMA3 expression. **E**–**G** GSEA-identified gene sets enriched in LAMA3 expression phenotype

**Fig. 11:**
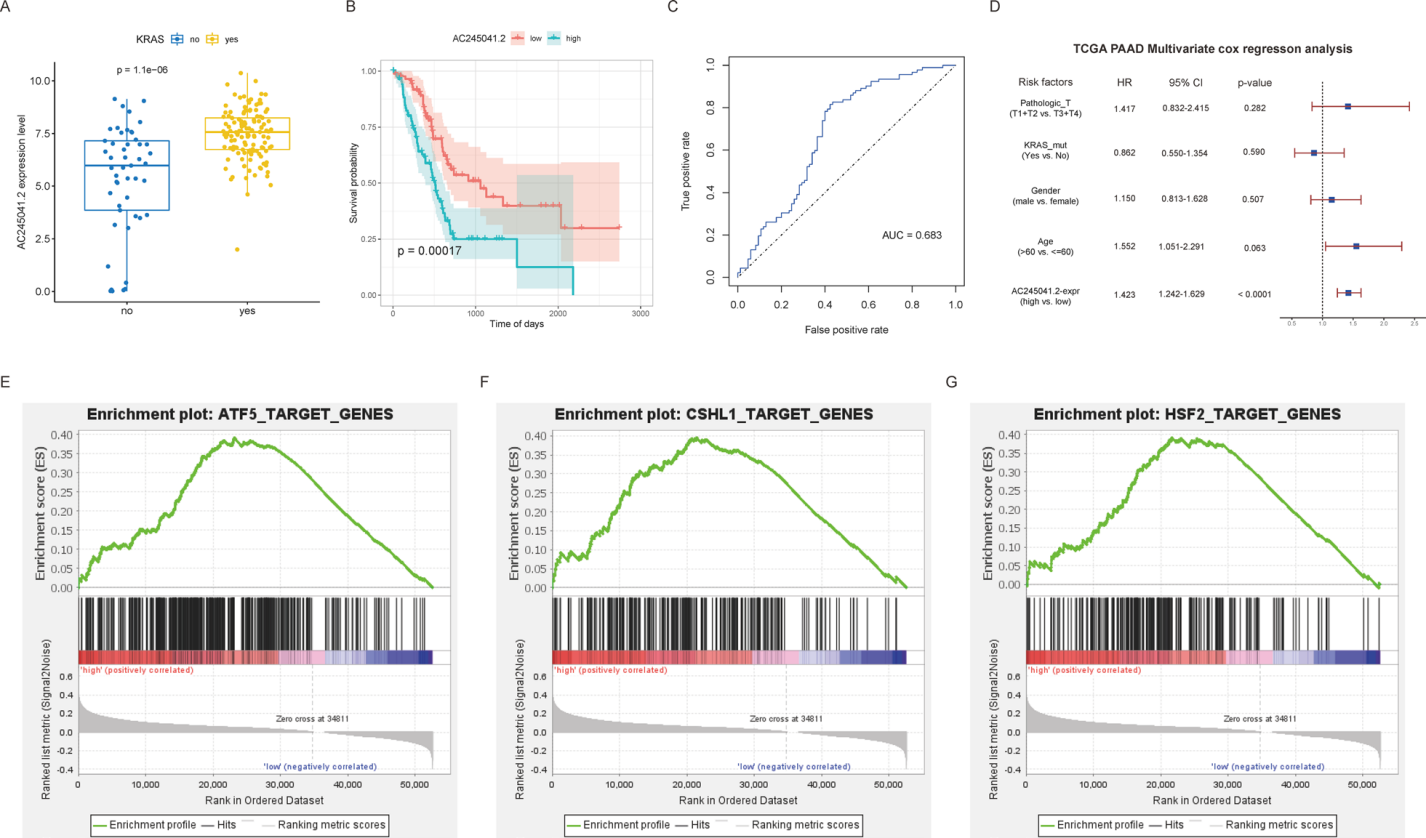
**A** Distribution of AC245041.2 expression in KRAS mutation groups. **B** KM curves to compare the overall survival between high-AC245041.2 and low-AC245041.2 samples. **C** ROC curve for prognosis based on AC245041.2 expression. **D** Multivariate Cox regression analysis based on AC245041.2 expression. **E**–**G** GSEA-identified gene sets enriched in LAMA3 expression phenotype

In the two risk models, Pearson’s correlation was adopted to analyze the correlation of each node in the two groups. It was observed that LAMA3 (mRNA) and AC245041.2 (lncRNA) were located in the two risk models, respectively, and the expression correlation of these two genes was the highest. The ROC curve was drawn to verify whether the expression of these two genes exerted independent prognostic effects. It was revealed that the AUC values of both genes were greater than 0.65, indicating that the expressions of both genes could be used as independent prognostic factors. Subsequently, the GSEA analysis of these two genes was performed, and the enrichment results were filtered using the thresholds of P-value < 0.05 and FDR < 0.25. The results revealed that the genes in the high-expression samples of the two genes were enriched in several pathways related to TFs, among which ATF5, CSHL1, NR1I2, SIPA1, HOXC13, HSF2, and HOXA10 were shared between the two groups (Figs. 10 and 11).

### Construction of the TF regulatory network associated with the hub mRNA–lncRNA regulatory axis

**Fig. 12:**
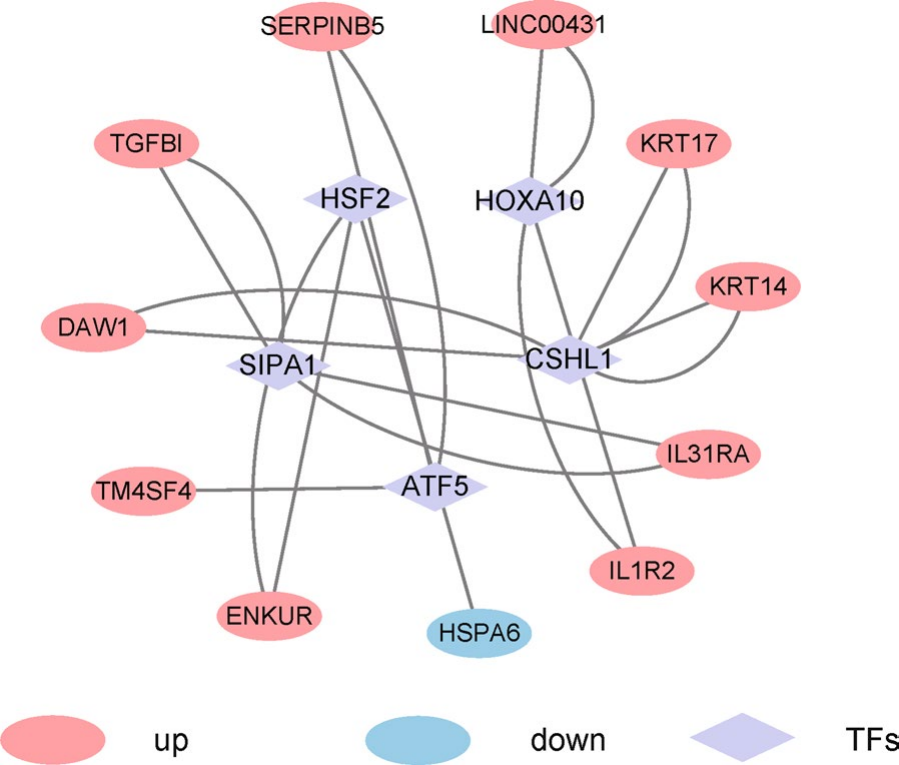
The mRNAs–lncRNA–TFs regulatory network

Considering that the high-expression samples of the two genes were enriched in several pathways related to TFs, a TF regulatory network associated with the hub mRNA–lncRNA regulatory axis was constructed based on LAMA3 and AC245041.2. According to the enrichment results for the genes in the LAMA3 high-expression samples, the core enrichment genes in the common TF-related pathways were screened out, and the differently expressed genes in the high and low expression groups of LAMA3 and AC245041.2 were obtained and integrated. Finally, the mRNA–lncRNA–TFs regulatory network was constructed (Fig. 12).

## Discussion

PAAD is one of the most malignant tumors of the digestive system, with an alarming mortality rate in both eastern and western nations (Ferlay et al. 2015; Ryan et al. 2014). Current clinical data demonstrate that surgery remains the only treatment option, even though only 20% of the patients survive 5 years after the pancreatic resection, and the benefits of chemotherapy are also limited (Dreyer et al. 2017; Zhu et al. 2018). Therefore, it is important to identify novel molecular biomarkers and elucidate the mechanisms underlying the occurrence and progression of PAAD. The importance of KRAS activation in PAAD was demonstrated in previous studies on sequencing in PAAD (Bailey et al. 2016). Approximately 90% of the pancreatic cancer genomes sequenced using targeted sequencing, whole-exome sequencing, or whole-genome sequencing exhibited carcinogenic KRAS. Activation of oncogenic KRAS in PAAD is associated with the occurrence and progression of tumors reportedly in several aspects, including the deregulation of key signal transduction pathways, metabolic changes, metastasis, and drug resistance (Mann et al. 2016).

However, the activation mechanism of mutant KRAS in PAAD has not been elucidated so far (Grant et al. 2016). Therefore, it is of great significance to identify the potential genes associated with KRAS mutation and PAAD prognosis. Mining relevant data from the TCGA–PAAD dataset might assist in identifying prognostic factors that could be involved in cancer occurrence and progression. In this context, the present study used the TCGA–PAAD dataset to identify the DE-lcnRNAs and DE-mRNAs between KRAS-mutant and KRAS-wildtype PAAD. The results of the enrichment analysis revealed that the enrichment mainly included the pathways for the modulation of chemical synaptic transmission, regulation of trans-synaptic signaling, signal release, synaptic membrane, neuronal cell body, channel activity, passive transmembrane transporter activity, and ion-gated channel activity, etc. Weighted co-expression network analysis of all the differentially expressed genes revealed the module with the strongest correlation with the prognostic traits. Multivariate Cox regression analysis enabled the construction of the prognostic risk models for lncRNAs and mRNAs to identify the hub differentially expressed genes associated with PAAD prognosis. Moreover, the analysis of the differences in the clinical characteristics between risk score1 and risk score2 revealed that the distribution of these differentially expressed genes was associated with the development of PAAD.

The following mRNAs were screened out with significant prognostic correlation: GLTPD2; RP1; MUC21; FAM83A; MYEOV; ZNF488; LAMA3 and LncRNA: AC068580.2; LINC01910; AC245041.2; AC107959.3; CASC8; UCA1; LINC00520; AL033384.1. A few of these genes have been identified previously in recent studies, among which the most reported one is UCA1, which is highly expressed in pancreatic cancer and is associated with the prognosis of this disease. UCA1 functions as a competing endogenous RNA (ceRNA) to increase the expression of KRAS via sponging miR-590–3p in pancreatic ductal adenocarcinoma; KRAS, in turn, promotes the UCA1 expression (Liu et al. 2019). Zhou et al. have suggested that UCA1 promotes proliferation, invasion, and migration, as well as the inhibition of apoptosis, in pancreatic cancer cells, through the downregulation of miR-96 and upregulation of FoxO3 (Zhou et al. 2018). Certain scholars have reported that UCA1 is highly expressed in the exosomes derived from hypoxic pancreatic cancer cells and could be transferred to human umbilical vein endothelial cells via these exosomes, which suggests that hypoxic exosomal UCA1 might promote angiogenesis and tumor growth through the miR-96–5p/AMOTL2/ERK1/2 axis (Guo et al. 2020). Recent studies have reported that MYEOV could be a potential prognostic biomarker and therapeutic target of pancreatic ductal adenocarcinoma. For instance, Liang et al. believed that MYEOV promoted the expression of hairy/enhancer of split homolog–1, a SOX9 target gene, by enhancing the SOX9 DNA-binding ability of the HES1 enhancer and that HES1 knockdown partially abrogated the oncogenic effect of MYEOV (Liang et al. 2020). Tang et al. reported that the high expression of MYEOV promoted glycolysis in the tumor cells in pancreatic ductal adenocarcinoma, which was validated in cellular assays (Tang et al. 2020). In addition, the FAM83A gene was reported to be amplified in several human cancers, while silencing FAM83A in related cancer cell lines inhibited the activation of the WNT/β-catenin and TGF-β signaling pathways besides reducing tumorigenicity (Zhang et al. 2019; Liu et al. 2020). Chen demonstrated that FAM83A overexpression significantly promoted the cancer stem cell-like characteristics and chemotherapy resistance of tumor cells in vitro as well as in vivo in the mouse models of pancreatic cancer, while FAM83A inhibition reduced the drug resistance of tumor cells (Chen et al. 2017). Kim analyzed the differential expression of genes between normal pancreas tissues and PAAD tissues using LASSO regression analysis to construct the prognostic gene expression model, which revealed LAMA3, E2F7, IFI44, SLC12A2, and LRIG1 as the potential drug targets in PAAD treatment (Kim et al. 2019). Yang used an online public database to evaluate the mRNA expression and the prognostic value of the laminin subunits in pancreatic ductal adenocarcinoma tissues; the author reported that LAMA3 and LAMC2 were positively correlated with the amount of pancreatic ductal adenocarcinoma blood and were, therefore, considered potential therapeutic targets and prognostic markers for pancreatic ductal adenocarcinoma (Yang et al. 2019). In the present study, LAMA3 (mRNA) and AC245041.2 (lncRNA), the genes that demonstrated the highest correlation, were identified in the two risk models, and both the genes were highly expressed in KRAS-mutant PAAD and could, therefore, be used as independent prognostic factors. The genes in the samples with high expression of LAMA3 were enriched in several pathways related to transcription factors. The core enrichment genes in the common TF-related pathways were collated, and the mRNA–lncRNA–TFs regulatory network was constructed, which might be closely associated with the prognosis of PAAD.

## Conclusion

In the present study, hub lncRNAs and mRNAs associated with KRAS mutation and PAAD prognosis were identified through comprehensive bioinformatics analysis. In addition, an mRNA–lncRNA–TFs regulatory network was constructed. The findings of the present study would deepen the understanding of the pathogenesis of KRAS-mutant PAAD and provide clues and novel insights for further research in this regard.

## Supplementary Information

**Additional file 1.** .Workflow chart of this study.

## Data Availability

The data used to support the findings of this study are included in the article.
