# Supplementary figures and images for: High expression of LAMA3/AC245041.2 gene pair associated with KRAS mutation and poor survival in pancreatic adenocarcinoma: a comprehensive TCGA analysis
Source: Mol Med. 2021 Jun 16;27:62. doi: 10.1186/s10020-021-00322-2 (PMC8207728; doi:10.1186/s10020-021-00322-2)

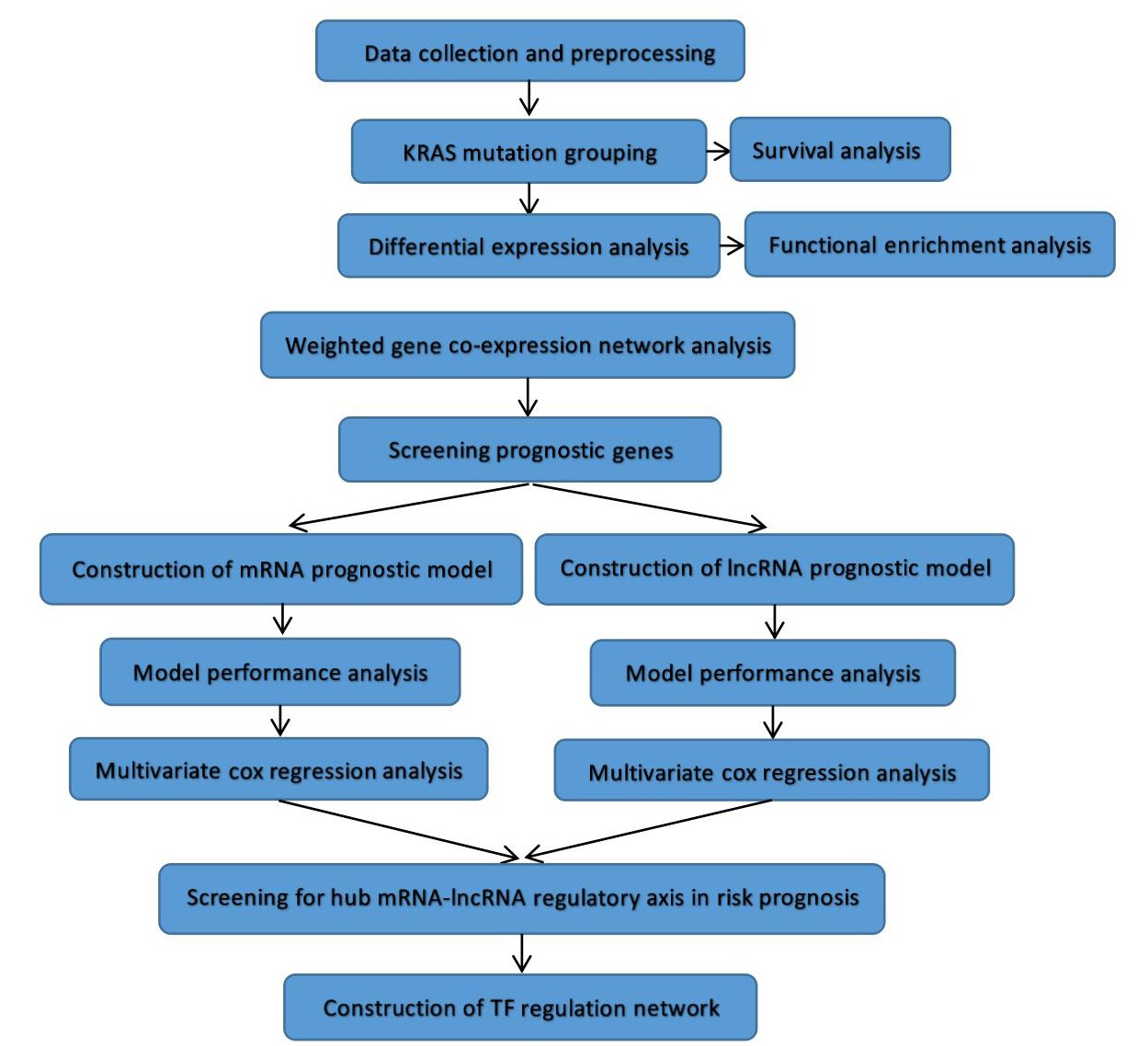

Supplement: Supplementary file 1 — Additional file 1. .Workflow chart of this study. [file 10020_2021_322_MOESM1_ESM.tif]
